# An Assessment of the Dimensionality and Factorial Structure of the Revised Paranormal Belief Scale

**DOI:** 10.3389/fpsyg.2017.01693

**Published:** 2017-09-26

**Authors:** Kenneth Drinkwater, Andrew Denovan, Neil Dagnall, Andrew Parker

**Affiliations:** Psychology, Manchester Metropolitan University, Manchester, United Kingdom

**Keywords:** belief in the paranormal, confirmatory factor analysis, bifactor model, revised paranormal belief scale, composite reliability

## Abstract

Since its introduction, the Revised Paranormal Belief Scale (RPBS) has developed into a principal measure of belief in the paranormal. Accordingly, the RPBS regularly appears within parapsychological research. Despite common usage, academic debates continue to focus on the factorial structure of the RPBS and its psychometric integrity. Using an aggregated heterogeneous sample (*N* = 3,764), the present study tested the fit of 10 factorial models encompassing variants of the most commonly proposed solutions (seven, five, two, and one-factor) plus new bifactor alternatives. A comparison of competing models revealed a seven-factor bifactor solution possessed superior data-model fit (CFI = 0.945, TLI = 0.933, IFI = 0.945, SRMR = 0.046, RMSEA = 0.058), containing strong factor loadings for a general factor and weaker, albeit acceptable, factor loadings for seven subfactors. This indicated that belief in the paranormal, as measured by the RPBS, is best characterized as a single overarching construct, comprising several related, but conceptually independent subfactors. Furthermore, women reported significantly higher paranormal belief scores than men, and tests of invariance indicated that mean differences in gender are unlikely to reflect measurement bias. Results indicate that despite concerns about the content and psychometric integrity of the RPBS the measure functions well at both a global and seven-factor level. Indeed, the original seven-factors contaminate alternative solutions.

## Introduction

Opinion polls and surveys consistently report that belief in the paranormal is widespread within modern society. Specifically, they indicate that a substantial proportion of the general population believe in the existence of supernatural powers and forces. Illustratively, the 2005 Gallup survey (comprising 1,002 telephone interviews with American adults) found that 73% of Americans expressed belief in paranormal phenomenon (Moore, 2005). This was especially true of extrasensory perception (ESP) (41%) and haunting (37%). The overall figure was similar to an earlier 2001 Gallup survey (Newport and Strausberg, 2001), which reported that the majority of the American population (76%) endorsed at least one paranormal belief. Compared with the prior 1990 Gallup Poll (Gallup and Newport, 1990), the 2001 survey demonstrated an increase in belief of more than five percentage points for several paranormal phenomena (haunted houses, ghosts, witches, communicating with the dead, psychic or spiritual healing, extra-terrestrial beings visiting earth and clairvoyance; the power of the mind to know the past and predict the future). Only belief in possession by the devil demonstrated a significant downturn.

MORI (Market and Opinion Research International) polls in Britain report comparable high levels of belief. The 2007 Survey on Beliefs (involving telephone interviews with a representative quota sample of 1,005 adults) found high endorsement of fate (62%), souls (62%) and premonitions (58%) (MORI, 2007). These figures were congruent with a previous 1998 MORI Paranormal Survey, which observed high endorsement of Premonitions/ESP (64%) (MORI, 1998). Collectively, Gallup and MORI polls evidence that belief in the paranormal is prevalent within contemporary society. This reflects the socially important nature and relevance of supernatural phenomena and explains/justifies sustained academic interest in the topic (Houran et al., 2001).

Alongside incidence of paranormal belief, researchers focus also on belief predictors. Hence, there is an established research tradition concerned with the study of correlates, which considers the psychological and socio-cultural foundations of paranormal belief (Lange et al., 2000). Correspondingly, articles referring to belief in the paranormal feature prominently within journals from a range of psychological sub-disciplines (e.g., personality, individual differences, cognitive, psychopathology, etc.). Whilst investigators have employed a range of measures to assess belief in the paranormal, the majority of work uses either the Revised Paranormal Belief Scale (RPBS) (Tobacyk, 2004), or the Australian Sheep-Goat Scale (ASGS) (Thalbourne and Delin, 1993; Wiseman and Watt, 2006). The RPBS because of its breadth, multidimensionality and preponderance in general psychological literature is the focus of the present paper. Indeed, the RPBS is the most widely used measure of paranormal belief (Goulding and Parker, 2001).

The ASGS in comparison possesses a narrower focus. It centers on traditional core paranormal concepts (extra-sensory perception, psychokinesis and life after death) and typically acts as a unitary, general index of paranormality (belief in psychic ability) (Thalbourne and Delin, 1993; Wiseman and Watt, 2006). The ASGS by virtue of emphasis and nature has historically featured more prominently within parapsychological literature.

The original Paranormal Belief Scale (PBS) (Tobacyk and Milford, 1983) arose from a factor analysis of a 61-item pool administered to 391 college students at Louisiana Tech University. Items sampled as wide a range of paranormal beliefs as possible and comprised questions modified from existing assessment instruments and newly devised statements. Conceptual coherence, in the absence of an agreed definition of the paranormal derived from implementation of three criteria (see Braude, 1978; Alcock, 1981); (a) current science and inexplicability of phenomena, (b) explicability requires major revision of the basic limiting principles of science (Broad, 1953), and (c) incompatibility with conventional notions of reality.

Factor analysis revealed seven independent factors (Traditional Religious Beliefs, Psi Beliefs, Witchcraft, Superstition, Spiritualism, Extraordinary Lifeforms and Precognition). All possessed clear, consistent structures and supported the notion that belief in the paranormal was a multidimensional construct. Prior to PBS construction, predetermined criteria specified clearest factor markers. Specifically, inclusion required that: (a) each marker possessed the largest loading on the relevant factor, and (b) the marker clearly reflected the factor theme. This process produced a 25-item scale derived from empirical investigation of belief via responses from a college sample, which represented separate paranormal dimensions. Further evaluation of the scale (Tobacyk and Milford, 1983), using 424 undergraduates, produced descriptive statistics and established the psychometric integrity of the PBS (convergent and discriminant properties).

Tobacyk reviewed the PBS (see Tobacyk, 1988, 2004) and developed the Revised Paranormal Belief Scale (RPBS). This involved contextualizing the nature of the RPBS, elucidating it as a measure of paranormal and religious beliefs, which facilitates examination of beliefs and their implications for spirituality. Adaptations to the PBS were: (1) implementation of a seven-point rather rating scale (PBS used a five-point scale); (2) development of a new Precognition subscale, item endorsement did not accurately reflect belief in precognition; and (3) modifications to the Witchcraft and Extraordinary Lifeforms subscales. Enhancements were designed to improve subscale reliability and validity (specifically, improve Western cross-cultural reliability) and lessen range restriction. Amendments resulted in the publication of the enhanced 26-item RPBS.

The scale has not been without criticism. Specifically, concerns and debate centered on the definition of paranormality employed to determine subject matter, item orthogonality (Tobacyk and Thomas, 1997), and factorial structure (Lawrence, 1995a,b; Tobacyk, 1995a,b; Lawrence and De Cicco, 1997; Lawrence et al., 1997, 1998). This discussion sits largely outside the remit of the present paper. However, the proposal of different factorial solutions, particularly the five and two-factor alternatives is important.

Particularly, Lawrence (1995a) demonstrated that an oblique five-factor model (Traditional Religious Belief, Psychic Beliefs, Superstition, Witchcraft and Anomalous Natural Phenomena) produced superior fit to the frequently cited Big Orthogonal Seven model (BOS) (see Lawrence et al., 1997). The value of the BOS is questionable because Tobacyk and Milford (1983) did not advance a mathematically based orthogonal model. They noted that subscales represented relatively independent dimensions (orthogonal), but were aware that cross-factor loadings factor did not advocate an uncorrelated subscale structure. Later, Tobacyk and Thomas (1997) suggested that a mixture of orthogonal and oblique relationships would most effectively represent the subfactors of the RPBS.

Following factor analysis of data from a sample of 560 Australian adults and removal of items showing pervasive differential item functioning, Lange et al. (2000) advocated a two-factor structure. This comprised New Age Philosophy (NAP; 11 items assessing largely psi, spiritualism and precognition) and Traditional Paranormal Beliefs (TPB; 5 items measuring traditional religious belief and witchcraft). These factors serve different functions. NAP instills a sense of control over external events at an *individual* level (Irwin, 1992), whilst TPB maintains control over external events on a *social* level (Goode, 2000). Hence, personal experiences potentially reinforce NAP and culture TPB. The two-factor model has appeared in several papers, however, many researchers still use the RPBS as a general measure of belief in the paranormal, or the original subscales as independent measures.

Hartman (1999) using two statistical procedures (the minimum average partial and parallel analysis criteria) determined that the RPBS contained only four latent variables (Psi, Traditional Religion, Superstition, and Witchcraft) rather than the commonly used seven (Tobacyk and Milford, 1983) or five factors (Lawrence and De Cicco, 1997; Lawrence et al., 1997). Whilst, Hartman's approach offers useful insights into the factorial structure of the RPBS the four-factor model lacks conceptual clarity and has never been widely implemented. For instance, the third factor labeled Superstition comprises items measuring precognition (specifically astrology) and extraordinary life forms (belief in Abominable snowman of Tibet and the Loch Ness monster). For these reasons, Hartman's (1999) solution is only included within the present paper for the sake of completeness.

Since its development, the RPBS has featured in myriad papers as indexed by articles on the Web of Science. Publications span psychological domains (cognitive, individual differences, psychopathology, etc.). Within these studies, paranormal belief researchers have scored the RPBS in a variety of ways. Particularly, overall level of paranormal belief (e.g., Wolfradt, 1997; Hergovich, 2003; Aarnio and Lindeman, 2005; Darwin et al., 2011), the seven original factors (e.g., Peltzer, 2003) and the two-factor structure (e.g., Dagnall et al., 2017b,a). Whilst, correlations reveal close correspondence between the different solutions it is important to know which solution best fits data. This is vital because the models place different theoretical emphasis on belief. Specifically, adoption of single factor scores assumes that belief in the paranormal is a unitary construct and that the RPBS acts as an overall index of paranormal belief. Seven and five-factor solutions derive from the notion that belief in the paranormal is comprised of conceptually distinct, but related factors. Acceptance of the notion that belief in the paranormal is multidimensional has facilitated the criticism that the finite number of items contained within the RPBS cannot adequately sample the paranormal belief domain (Lawrence, 1995a; Hartman, 1999). This notion ignores the fact there are innumerable examples of scales with similar numbers of items that assess constructs and contain multiple related but independent factors (e.g., Self-Compassion Scale, Neff, 2003).

Contrastingly, the two-factor structure endorsed by Lange et al. (2000) focuses on belief function rather than content. It derived from the notion that measurement instruments in order to assess dimensionality must be free of differential item functioning (Lange et al., 2000). Hence, the RPBS was Rasch scaled to ensure that item responses reflected construct endorsement rather than characteristics, such as age or gender. Whilst, this is an important consideration many articles have continued to employ the conventional scoring methods, using the RPBS as a global measure of paranormal belief, and/or referred to the original seven-factor solution.

In addition to disagreement concerning the RPBS factorial composition, the dimensionality of the RPBS lacks confirmation. Tobacyk and Milford (1983) in their original analysis concluded that the RPBS was multidimensional. However, this supposition did not derive from confirmatory factor analysis (CFA). CFA is a powerful statistical technique that enables researchers to test the adequacy of theoretically plausible models via specification of underlying structure (see Bollen, 1989). Furthermore, subsequent work using CFA (e.g., Lawrence, 1995a; Lawrence et al., 1997) failed to test explicitly RPBS multidimensionality, and Irwin (2009) emphasizes that the notion of the RPBS being multidimensional requires further confirmatory evidence. This issue is problematic because numerous studies use RPBS total scores to assess belief in the paranormal (see Wolfradt, 1997; Hergovich, 2003; Aarnio and Lindeman, 2005; Darwin et al., 2011). A CFA-based test of multidimensionality vs. unidimensionality is achievable via bifactor modeling. Bifactor modeling determines subscale viability and indicates whether a measure represents a single dimension (Reise et al., 2010). Explicitly, after controlling for the influence of a general factor, bifactor modeling specifies item loading strength and subscale reliability. Strong item loadings and reliability coefficients for subscales relative to a general factor indicates that the data is likely to be multidimensional. Otherwise, subscales are unnecessary and a general factor underpins the measure.

The paranormal belief scale has also been adapted for use within different countries (e.g., China, Shiah et al., 2010; Spanish, Diaz-Vilela and Alvarez-Gonzalez, 2004; France, Bouvet et al., 2014). Analysis of modified versions has also produced alternative factorial structures. For instance, Utinans et al. (2015) produced a Latvian Version of the RPBS, which yielded a six-factor structure (Magical Abilities, Psychokinesis, Traditional Religious Belief, Superstition, Spirit Travel, and Extraordinary Life Forms). Additionally, a study using undergraduate students from the University of Zagreb, Croatia (Mikloušić et al., 2012), produced a previously unreported three-factor solution. This comprised General Paranormal Belief (mostly Psi and Spiritualism subscale items, with some Precognition, Witchcraft and Extraordinary Lifeforms items); Traditional Religious Belief (Traditional Religious Beliefs scale items); and Rituals and Practices (Superstition subscale items and the remaining Precognition, Witchcraft and Extraordinary Lifeforms subscale items).

Collectively, these studies support the notion that factor composition and item fit vary as a function of socio-cultural context (Tobacyk and Thomas, 1997; Bouvet et al., 2014). At the general level, cultural differences are evident. Illustratively, a cross-cultural comparison between university students from Finland and America revealed that American students scored generally higher across measures of paranormal belief (Traditional Religious Belief, Superstition, Witchcraft, and Extraordinary Lifeforms; Tobacyk and Pirttilä-Backman, 1992). At the item level, adaptation is required to ensure that items are relevant. For instance, Dag (1999) revised Extraordinary Lifeform items for use with a Turkish sample (e.g., Loch Ness monster of Scotland replaced by the Van Lake monster, and exchanged the snowman of Tibet with the wolfman).

### The present study

The current study examined psychometric concerns about RPBS factor composition and dimensionality. Consideration of these issues was vital because conceptual stance and/or researcher preference determines RPBS scoring (i.e., total, seven-factor and two-factor), data analysis and interpretation. Thus, a comprehensive evaluation of RPBS latent structure was undertaken. This involved examination of several previously proposed models: two-factor model of Lange et al. (2000), one-factor solution (as a null model), five-factor and seven-factor solutions (Tobacyk and Milford, 1983; Lawrence, 1995a; Lawrence et al., 1997; Tobacyk and Thomas, 1997). Alongside these, bifactor variants of five-factor and seven-factor models were tested.

In summary, RPBS evaluation was necessary for several reasons. Firstly, to address how many subfactors best represent the RPBS and, in turn, to assess whether the RPBS functions as a multidimensional or general paranormal factor measure (Tobacyk and Milford, 1983; Irwin, 2009). Secondly, studies frequently employ total RPBS scores in conjunction with individual factors (e.g., Wolfradt, 1997; Hergovich, 2003; Aarnio and Lindeman, 2005; Darwin et al., 2011) and conceptualize belief in the paranormal as a latent factor (see Hergovich et al., 2008; Darwin et al., 2011). However, studies have failed to test the adequacy of these assumptions by including multidimensionality vs. unidimensionality within a single analysis. The inclusion of bifactor modeling determined whether scores from the RPBS best represented a single dimension or several specific factors. Since factors within bifactor models must not correlate, the technique allows an unambiguous assessment of scores on a general dimension without the influence of specific factors (Reise et al., 2007). Simultaneously, bifactor models determine whether specific facets exist after partialling out a general factor (Chen et al., 2012). In the context of the RPBS, bifactor modeling enabled an assessment of dimensionality and solution adequacy. Specifically, comparison of competing models clarified the latent structure of the RPBS.

## Methods

### Participants

Several data sets containing completed RPBS measures were merged to produce a large heterogeneous sample (*N* = 3,764). These straddled the period between January 2008 and January 2017 (see ethics section). The aggregated sample comprised data from several published studies (e.g., Dagnall et al., 2016, 2017a) and new samples. This sampling approach was similar to that employed by Roets and Van Hiel (2011), who produced an amalgamated sample from previous studies in order to validate their Need for Closure Scale. Lange et al. (2000) in their top-down purification the RPBS also employed a similar approach; they used data from several studies collected over a 10 year period.

Consideration of the sample revealed 2,495 participants were students and 1,269 non-students. Of these, 1,069 (28%) respondents were male and 2,695 (72%) female. Within groups, 17% of students were male and 83% females, whilst 36% of non-students were male and 64% female. The mean age for males was 29.45 years (*SD* = 12.23, range of 18–79 years) and the mean age for females was 26.67 years (*SD* = 10.89, range of 18–78 years). The only exclusion criterion was that respondents had to be at least 18 years of age. To prevent multiple responses, instructions routinely ask respondents to indicate whether they have participated within similar studies.

### Measure

The only measure analyzed within this study was the 26-item Revised Paranormal Belief Scale (RPBS). Within the RPBS, questions appear as statements (e.g., “There is a devil”; Tobacyk, 1988, 2004). Participants respond to each item via completion of a seven-point Likert scale (answers range from 1 = strongly disagree to 7 = strongly agree). Items index seven facets of belief: Precognition, Psi Belief, Traditional Religious Belief, Spiritualism, Witchcraft, Superstition and Extraordinary Lifeforms. Previous research reports that the RPBS possesses satisfactory reliability and adequate validity (Tobacyk, 2004). Hence, researchers generally regard the RPBS as a satisfactory measure of belief in the paranormal (Tobacyk, 2004). Some critics, however, question the psychometric properties of individual dimensions and forward alternative solutions (see introduction; Cardeña et al., 2015). Subsequently, Lange et al. (2000) purified the RPBS. This process identified a two-factor solution centered on belief function (individual vs. social). This includes New Age Philosophy (NAP) (11-items assesses belief in psi and survival of bodily death) and Traditional Paranormal Belief (TPB) (5-items measure belief in concepts, such as the devil, witchcraft, heaven and hell) (Cardeña et al., 2015). At the individual level, NAP imparts control over external events (Irwin, 1992), whilst TPB regulates social/cultural factors (Goode, 2000).

In the current study, Cronbach alpha reliability for the total scale was high (α = 0.93). For the seven subscales, alpha reliability was good for Traditional Religious Belief (α = 0.88), Witchcraft (α = 0.80), Psi Beliefs (α = 0.83), Superstition (α = 0.83), Spirituality (α = 0.83), and Precognition (α = 0.86). For Extraordinary Lifeforms, however, alpha was below the recommended threshold of 0.7 (α = 0.54). For the two subscales identified by Lange et al. (2000), alpha reliability was good, NAP (α = 0.86) and TPB (α = 0.81).

### Procedure

Respondents within the studies underpinning the amalgamated data set undertook the same general procedure (these studies centered on anomalous beliefs, cognitive-perceptual personality factors and decision-making). Prior to participating potential respondents read the study background information, this stated the nature of the study and outlined ethical procedures. Respondents agreeing to participate indicated informed consent and received the materials booklet. Instructions asked respondents to carefully read questions, answer all questions, take their time and complete items in an open and honest manner. The order of questionnaires typically rotated across sections. Respondents provided also demographic information (preferred gender, age, etc.).

### Ethics

The researchers obtained ethical approval for the studies as part of two successful grant proposals (September 2008 and 2010) and three unsuccessful bids (September 2012, 2014, and 2016). In the case of each application, The Director of the Research Institute for Health and Social Change (Faculty of Health, Psychology and Social Care) within Manchester Metropolitan University ratified the project (methodological and ethical). This is the necessary level of ethical clearance for projects rated as “routine.” Furthermore, it is a university condition that research proposals are peer-reviewed by members of the Professoriate (or equivalent) prior to submission. This includes ethical scrutiny and gaining clearance in principal. Additionally, the Head of the Psychology Department must sanction research projects. Formal submission to a university ethics panel beyond this process is not an institutional requirement for routine studies.

### Analysis

To examine comprehensively the latent structure of the RPBS confirmatory factor analysis (CFA) tested the adequacy of ten competing models using AMOS 24. Table 1 provides a description of each model.

**Table 1:** Competing factor models of the revised paranormal belief scale.

**Model type**	**Description and item allocation**
Two-factor oblique (Lange et al., 2000)	Two correlated factors: Traditional Paranormal Beliefs (items 8, 17, 22, 24, 26), and New Age Philosophy (items 2, 3, 5, 7, 9, 12, 14, 16, 19, 21, 23)
One-factor	All 26 RPBS items specified to load on a single factor
Five-factor oblique (Lawrence, 1995a)	Mixture of orthogonal and oblique relationships among five factors: Traditional Religious Belief (items 1, 8, 15, 22), Psychic Beliefs (items 2, 5, 9, 12, 16, 19, 21, 23, 25, 26), Superstition (items 4, 11, 18), Witchcraft (items 3, 10, 17, 24), and Anomalous Natural Phenomena (items 6, 7, 13, 14, 20)
Five-factor oblique (Lawrence et al., 1997)	Same as Lawrence (1995a), but with different factor correlations
Five-factor orthogonal (Lawrence et al., 1997)	Same factor composition as Lawrence (1995a), but with orthogonal relationships specified among factors
Five-factor bifactor	Six factors: Traditional Religious Belief (items 1, 8, 15, 22), Psychic Beliefs (items 2, 5, 9, 12, 16, 19, 21, 23, 25, 26), Superstition (items 4, 11, 18), Witchcraft (items 3, 10, 17, 24), Anomalous Natural Phenomena (items 6, 7, 13, 14, 20), and RPBS-Total (all scale items)
Seven-factor orthogonal (Tobacyk and Milford, 1983)	Seven orthogonal factors: Traditional Religious Belief (items 1, 8, 15, 22), Psi Beliefs (items 2, 9, 16, 23), Superstition (items 4, 11, 18), Witchcraft (items 3, 10, 17, 24), Spiritualism (items 5, 12, 19, 25), Precognition (items 7, 14, 21, 26), and Extraordinary Lifeforms (items 6, 13, 20)
Seven-factor mixed model (Tobacyk and Thomas, 1997)	Same as Tobacyk and Milford (1983), but with a mixture of orthogonal and oblique relationships among factors
Seven-factor oblique (Lawrence et al., 1997)	Same factor composition as Tobacyk and Milford (1983), but with oblique relationships specified among factors
Seven-factor bifactor	Eight factors: Traditional Religious Belief (items 1, 8, 15, 22), Psi Beliefs (items 2, 9, 16, 23), Superstition (items 4, 11, 18), Witchcraft (items 3, 10, 17, 24), Spiritualism (items 5, 12, 19, 25), Precognition (items 7, 14, 21, 26), Extraordinary Lifeforms (items 6, 13, 20), and RPBS-Total (all scale items)

Consideration of a range of indices determined data-model fit. The chi-square (χ^2^) statistic examines the difference between the observed and expected covariance matrix. A non-significant result denotes good fit. However, chi-square is sensitive to sample size and with large samples often over-rejects good models. The Comparative Fit Index (CFI), the Incremental Fit Index (IFI) and the Tucker-Lewis Index (TLI) compare a proposed model with a null model, where variables are uncorrelated (McDonald and Ho, 2002). Values above 0.90 specify adequate fit (Hu and Bentler, 1999). The Root-Mean-Square Error of Approximation (RMSEA) is a noncentrality-based index that identifies the quantity of variance-covariance data not effectively predicted by a hypothesized model. The Standardized Root-Mean-Square Residual (SRMR) is the square root of the misfit between a model covariance matrix and a sample covariance matrix. For RMSEA, the 90% confidence interval (CI) was included. Values below 0.08 for RMSEA and SRMR advocate reasonable fit (Hu and Bentler, 1999).

For each model, consideration of Modification Indices (MI) revealed the degree to which a model chi-square improved if constrained parameters were free to covary. High MI values (i.e., above 25; Torres-Harding et al., 2012) pertaining to subfactor items were investigated. Byrne (2010) recommends avoidance of covarying within-item errors unless reasonable justification is present. Instances where error covariance is justifiable include when the parameters in question are characterized by non-random measurement error (e.g., method effects resulting from similarities in item content). Error covariance across subfactors was, however, not permissible given the differences in item content (Byrne, 2010). Akaike's Information Criterion (AIC) and the Expected Cross-Validation Index (ECVI) facilitated model comparison, with lower values indicating better fit.

The superior factor structure was subjected to invariance testing in relation to gender at the configural, metric, and scalar level. Configural invariance tests require the same factor structure to hold across the tested groups. For metric invariance, it is necessary for the factor loadings to be the same (invariant) across groups. Scalar invariance requires the intercepts to be invariant across groups. Satisfaction of scalar invariance testing suggests that mean comparisons across groups are valid and are not symptomatic of measurement bias. To determine invariance, Cheung and Rensvold (2002) recommend in addition to satisfactory model fit, that CFI values should not change by more than 0.02 between models. Similarly, due to its sensitivity chi-square is not recommended as an index for invariance in samples of 1,000 or greater (Brown, 2006). Lastly, composite reliability of the superior factor solution assessed the reliability of the RPBS.

## Results

Prior to analysis, data screening occurred and resulted in the removal of 20 extreme scores. This left a final sample of 3,744. The RPBS mean was 80.86 (*SD* = 28.56). Skewness and kurtosis values were within the recommended interval of −2 to +2 (Byrne, 2010; see Table 2). A comparison of gender scores revealed that women (*M* = 85.43, *SD* = 27.18) scored significantly higher in paranormal beliefs than men (*M* = 69.84, *SD* = 28.81), *t*(3,742) = 15.31, *p* < 0.001, *d* = 0.55 (medium effect). Inter-correlations between the seven subscales outlined in the original RPBS (Tobacyk, 1988) and among scale items were significant. Furthermore, there were no instances of multicollinearity, all inter-correlations were below 0.9 (Tabachnick and Fidell, 2001). A correlation above 0.9 was evident between New Age Philosophy (NAP) and RPBS-Total (two-factor solution). However, this was unsurprising given that a high proportion of RPBS-Total items comprise NAP.

**Table 2:** Descriptive statistics and intercorrelations for RPBS-Total and subscales (*N* = 3,744).

**Variable**	**Mean**	***SD***	**Skew**	**Kurtosis**	**1**	**2**	**3**	**4**	**5**	**6**	**7**	**8**	**9**	**10**
1. RPBS-total	80.86	28.56	0.12	−0.71		0.70^**^	0.62^**^	0.81^**^	0.84^**^	0.57^**^	0.78^**^	0.87^**^	0.89^**^	0.93^**^
2. Traditional religious belief	14.93	7.21	0.14	−0.97			0.36^**^	0.49^**^	0.44^**^	0.23^**^	0.40^**^	0.51^**^	0.82^**^	0.52^**^
3. Superstition	6.93	4.18	0.90	0.01				0.37^**^	0.55^**^	0.32^**^	0.40^**^	0.44^*^	0.46^**^	0.51^**^
4. Witchcraft	12.57	6.60	0.32	−0.86					0.60^**^	0.42^**^	0.64^**^	0.63^*^	0.83^**^	0.74^**^
5. Precognition	11.60	5.79	0.31	−0.85						0.43^**^	0.70^**^	0.75^**^	0.70^**^	0.87^**^
6. Extraordinary lifeforms	9.72	3.34	0.25	−0.19							0.45^**^	0.46^**^	0.40^**^	0.50^**^
7. Psi beliefs	11.80	5.67	0.44	−0.49								0.72^**^	0.63^**^	0.90^**^
8. Spirituality	13.02	6.55	0.74	0.47									0.68^**^	0.90^**^
9. Traditional paranormal beliefs	16.31	7.35	0.15	−0.81										0.76^**^
10. New age philosophy	32.63	14.48	0.39	0.01										

** *p < 0.001*.

CFA fit indices for the two-factor oblique model indicated unacceptable fit on all indices, but SRMR: χ^2^(103, *N* = 3,744) = 9,103.46, *p* < 0.001, CFI = 0.764, TLI = 0.725, IFI = 0.764, SRMR = 0.078, RMSEA = 0.153 (CI of 0.150–0.155). Modification indices revealed the presence of high error covariance between items 2 and 16, 5 and 12, 7 and 14, 7 and 21, 9 and 16, 8 and 22, 12 and 19. Allowing these error terms to correlate significantly improved fit, χ^2^ difference (8, *N* = 3,744) = 5,607.53, *p* < 0.001, resulting in acceptable fit on all indices, but TLI and RMSEA (see Table 3). Interestingly, all within-errors that were free to covary corresponded with the original RPBS factors.

**Table 3:** Fit indices for competing RPBS factor solutions.

**Model**	**χ2**	***df***	**CFI**	**TLI**	**IFI**	**SRMR**	**RMSEA (90% CI)**	**AIC**	**ECVI**
Two-factor oblique (Lange et al., 2000)	9,103.46^**^	103	0.764	0.725	0.764	0.078	0.153 (0.150–0.155)	9,201.46	2.45
Two-factor oblique (CE) (Lange et al., 2000)	3,495.93^**^	95	0.911	0.887	0.911	0.047	0.098 (0.095–0.101)	3,609.93	0.96
One-factor	20,941.65^**^	299	0.663	0.634	0.663	0.089	0.136 (0.134–0.137)	21,097.65	5.63
Five-factor oblique (Lawrence, 1995a)	12,979.34^**^	296	0.793	0.773	0.793	0.238	0.107 (0.105–0.109)	13,141.34	3.51
Five-factor oblique (Lawrence et al., 1997)	9,984.33^**^	292	0.842	0.824	0.842	0.160	0.094 (0.093–0.096)	10,154.33	2.71
Five-factor oblique (Lawrence et al., 1997) (CE)	5,550.38^**^	281	0.914	0.901	0.914	0.155	0.071 (0.069–0.072)	5,742.38	1.53
Five-factor orthogonal (Lawrence et al., 1997)	16,328.45^**^	299	0.739	0.716	0.739	0.303	0.120 (0.118–0.121)	16,484.45	4.40
Five-factor orthogonal (CE) (Lawrence et al., 1997)	12,022.89^**^	288	0.809	0.784	0.809	0.302	0.104 (0.103–0.106)	12,200.89	3.26
Five-factor bifactor	5,627.63^**^	273	0.913	0.896	0.913	0.051	0.072 (0.071–0.074)	5,835.63	1.55
Five-factor bifactor (CE)	4,860.48^**^	269	0.925	0.910	0.925	0.050	0.068 (0.066–0.069)	5,076.48	1.35
Seven-factor orthogonal (Tobacyk and Milford, 1983)	18,722.39^**^	299	0.700	0.673	0.700	0.338	0.128 (0.127–0.130)	18,878.39	5.04
Seven-factor orthogonal (CE) (Tobacyk and Milford, 1983)	17,199.78^**^	295	0.724	0.696	0.724	0.337	0.124 (0.122–0.125)	17,363.78	4.63
Seven-factor mixed (Tobacyk and Thomas, 1997)	7,624.62^**^	283	0.880	0.862	0.880	0.165	0.083 (0.082–0.085)	7,812.62	2.08
Seven-factor mixed (CE) (Tobacyk and Thomas, 1997)	5,746.01^**^	278	0.911	0.896	0.911	0.162	0.072 (0.071–0.074)	5,944.01	1.58
Seven-factor oblique (Lawrence et al., 1997)	6,077.63^**^	278	0.905	0.889	0.906	0.062	0.075 (0.073–0.076)	6,275.63	1.67
Seven-factor oblique (CE) (Lawrence et al., 1997)	4,154.49^**^	273	0.937	0.925	0.937	0.055	0.062 (0.060–0.063)	4,362.49	1.16
Seven-factor bifactor	4,371.34^**^	273	0.933	0.920	0.933	0.048	0.063 (0.062–0.065)	4,579.34	1.22
Seven-factor bifactor (CE)	3,669.80^**^	270	0.945	0.933	0.945	0.046	0.058 (0.056–0.060)	3,883.80	1.03

CE, correlated errors; χ2, chi-square goodness-of-fit statistic; df, degrees of freedom; CFI, Comparative Fit Index; TLI, Tucker-Lewis Index; IFI, Incremental Fit Index; SRMR, Standardized Root-Mean-Square Residual; RMSEA, Root-Mean-Square Error of Approximation; AIC, Akaike Information Criterion; ECVI, Expected Cross-Validation Index;

** *χ2 significant at p < 0.001*.

The one-factor model reported poor fit: χ^2^(299, *N* = 3,744) = 20,941.65, *p* < 0.001, CFI = 0.663, TLI = 0.634, IFI = 0.663, SRMR = 0.089, RMSEA = 0.136 (CI of 0.134–0.137). High error covariance was present in more than 50% of the items. Consequently, the solution did not allow for correlation between item errors (Byrne, 2010). The five-factor oblique model (Lawrence, 1995a) suggested unacceptable data fit on all indices: χ^2^(296, *N* = 3,744) = 12,979.34, *p* < 0.001, CFI = 0.793, TLI = 0.773, IFI = 0.793, SRMR = 0.238, RMSEA = 0.107 (CI of 0.105–0.109). In comparison, the modified five-factor oblique model (Lawrence et al., 1997) demonstrated improved fit: χ^2^(292, *N* = 3,744) = 9,984.33, *p* < 0.001, CFI = 0.842, TLI = 0.824, IFI = 0.842, SRMR = 0.160, RMSEA = 0.094 (CI of 0.093 to 0.096). However, data-fit remained unacceptable across indices. In addition, it was not possible to conduct chi-square difference between the five-factor models because of the orthogonal nature of the Anomalous Natural Phenomena factor (see Lawrence, 1995a).

MI values for the Lawrence et al. (1997) solution reported high error covariance for several items (items 1 and 22, 10 and 24, 6 and 13, 12 and 5, 19 and 25, 25, 21 and 26, 2, 9, and 16). Data-model fit significantly improved by permitting correlations of these error terms: χ^2^ difference (11, *N* = 3,744) = 4,433.95, *p* < 0.001, and resulted in acceptable fit on all indices but SRMR: χ^2^(279, *N* = 3,744) = 5,550.38, *p* < 0.001, CFI = 0.914, TLI = 0.901, IFI = 0.914, SRMR = 0.155, RMSEA = 0.071 (CI of 0.069–0.072). The five-factor orthogonal solution based on Lawrence et al. (1997) demonstrated poor fit on all indices: χ^2^(299, *N* = 3,744) = 16,328.45, *p* < 0.001, CFI = 0.739, TLI = 0.716, IFI = 0.739, SRMR = 0.303, RMSEA = 0.120 (CI of 0.118 to 0.121). Correlating error terms between items 1 and 15, 3, and 17, 6, and 13, 12, and 5, 19, and 25, 25, 21, and 16, 2, 9, and 16 significantly improved fit: χ^2^ difference (11, *N* = 3,744) = 4,305.56, *p* < 0.001, but this remained unsatisfactory.

The five-factor bifactor model reported acceptable fit on all indices, but TLI: χ^2^(273, *N* = 3,744) = 5627.63, *p* < 0.001, CFI = 0.913, TLI = 0.896, IFI = 0.913, SRMR = 0.051, RMSEA = 0.072 (CI of 0.071 to 0.074). High error covariance was evident for items 1 and 15, 3 and 17, 12 and 5, 25 and 26. Model fit significantly improved by permitting correlations among error terms for these items: χ^2^ difference (4, *N* = 3,744) = 767.15, *p* < 0.001. The orthogonal seven-factor model (Tobacyk and Milford, 1983) indicated unacceptable fit on all indices: χ^2^(299, *N* = 3,744) = 18,722.39, *p* < 0.001, CFI = 0.700, TLI = 0.673, IFI = 0.700, SRMR = 0.338, RMSEA = 0.128 (CI of 0.127–0.130). Allowing within-item errors between items 1 and 15, 3 and 17, 19 and 25, and 7 and 14 to correlate significantly improved fit: χ^2^ difference (4, *N* = 3,744) = 1,522.60, *p* < 0.001. However, data-model fit remained unsatisfactory.

To determine which subfactors should correlate (oblique vs. orthogonal) for the mixed seven-factor model (Tobacyk and Thomas, 1997), a two-stage process was applied. Firstly, an assessment of inter-item correlations was undertaken; all possessed significant relationships (i.e., *p* < 0.05). Next, based on subscales an examination of inter-correlations occurred (see Table 2). Where moderate relationships existed between subfactors (i.e., inter-correlations greater than 0.4; Evans, 1996) these were correlated in CFA. Accordingly, Traditional Religious Belief correlated with Witchcraft, Precognition, and Spiritualism. Superstition correlated with Precognition and Spiritualism. Witchcraft correlated with all subfactors, but Superstition. Precognition and Spiritualism correlated with all subfactors. Extraordinary Lifeforms and Psi Beliefs correlated with one another, Witchcraft, Precognition, and Spiritualism. Results suggested unacceptable fit on all indices: χ^2^(283, *N* = 3,744) = 7,624.62, *p* < 0.001, CFI = 0.880, TLI = 0.862, IFI = 0.880, SRMR = 0.165, RMSEA = 0.083 (CI of 0.082 to 0.085). Allowing within-item error correlations between items 1 and 22, 10 and 24, 5 and 12, 7 and 14, and 21 and 26 significantly improved fit, χ^2^ difference (5, *N* = 3,744) = 1,878.61, *p* < 0.001, and fit was satisfactory on all indices, but TLI and SRMR. The seven-factor oblique solution reported acceptable fit on all indices, but TLI: χ^2^(278, *N* = 3,744) = 6,077.63, *p* < 0.001, CFI = 0.905, TLI = 0.889, IFI = 0.905, SRMR = 0.062, RMSEA = 0.075 (CI of 0.073–0.076). Model fit significantly improved by permitting within-item errors between items 1 and 22, 10 and 24, 5 and 12, 7 and 14, 21 and 26 to correlate: χ^2^ difference (5, *N* = 3,744) = 1,923.13, *p* < 0.001.

**Figure 1:**
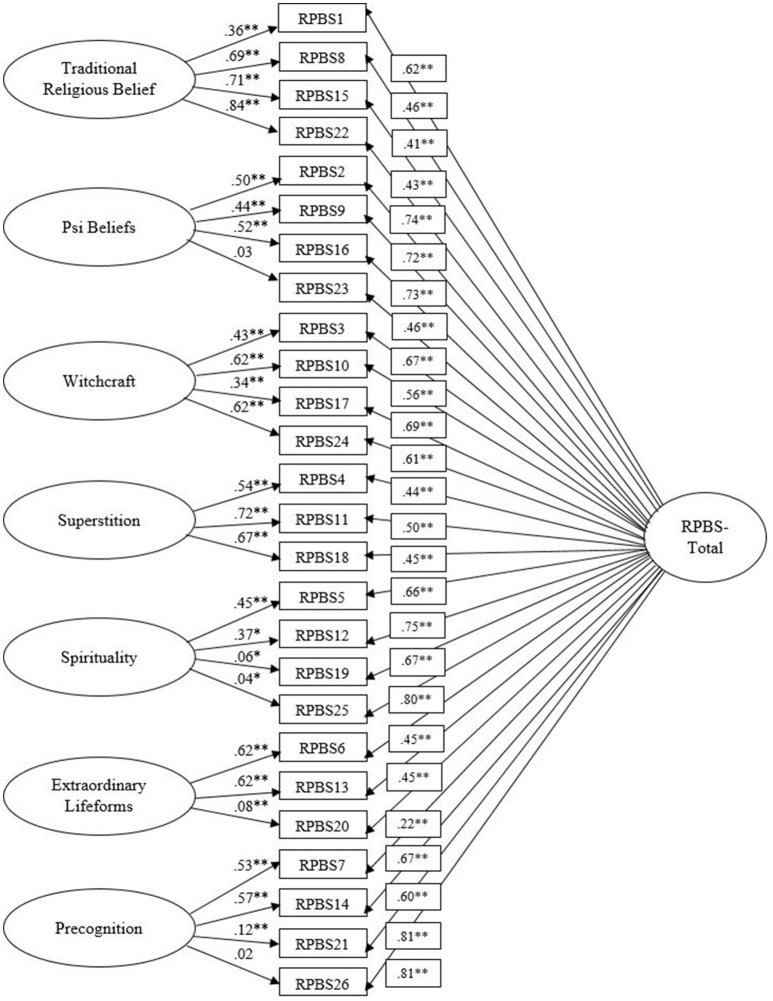
Seven-factor bifactor RPBS model. Latent variables are represented by ellipses; measured variables are represented by rectangles; error is not shown, but was specified for all variables. Error covariances between RPBS1 and RPBS15, RPBS3 and RPBS17, RPBS21 and RPBS26 are not shown but were included in the analysis. ^*^p < 0.05; ^**^p < 0.001.

The seven-factor bifactor model reported acceptable data-model fit on all indices: χ^2^(273, *N* = 3,744) = 4,371.34, *p* < 0.001, CFI = 0.933, TLI = 0.920, IFI = 0.933, SRMR = 0.048, RMSEA = 0.063 (CI of 0.062–0.065). Allowing within-item errors between items 1 and 15, 3 and 17, and 21 and 26 to correlate significantly improved fit: χ^2^ difference (3, *N* = 3,744) = 701.53, *p* < 0.001. Overall, the seven-factor bifactor model demonstrated superior fit in comparison with the other factor models, as evidenced by superior fit and lower AIC and ECVI statistics^1^ (see Table 3). Parameter estimates for the seven-factor bifactor solution further support the appropriateness of this model, as all factor loadings for RPBS-Total were statistically signissficant and exceeded the minimum threshold of 0.32 (Tabachnick and Fidell, 2001), with the exception of item 20 (loading of 0.22) (see Figure 1). The relative strength of the factor loadings for the subscale factors and the general factor provide important information in relation to the appropriateness of including subscales when scoring the RPBS. Specifically, when items load more highly on subscales than a general factor, this suggests that a measure comprises distinct subscales. When items load more highly on a general factor, this indicates that total scores are valid and that an underlying construct underpins the measure (Reise et al., 2010). In terms of the subscales, some item loadings were non-significant (items 23 and 26). However, the majority of items loaded higher than 0.32. These results provide tentative support for a general paranormal belief dimension and the existence of separate subscales.

Tests for invariance were subsequently performed in relation to gender. The configural invariance test revealed satisfactory fit, CFI = 0.925, TLI = 0.911, IFI = 0.925, SRMR = 0.048, RMSEA = 0.047 (CI of 0.046 to 0.048). A test for metric invariance also indicated satisfactory fit, CFI = 0.926, TLI = 0.919, IFI = 0.927, SRMR = 0.055, RMSEA = 0.045 (CI of 0.044–0.046), with a CFI difference less than the threshold of 0.02, confirming invariance at configural and metric stages across gender. The scalar invariance test reported acceptable fit, CFI = 0.917, TLI = 0.912, IFI = 0.917, SRMR = 0.055, RMSEA = 0.047 (CI of 0.046–0.048), with a CFI difference <0.02, supporting strong factorial invariance.

Many researchers regard internal reliability as a critical factor for determining a measure's suitability. Composite reliability, which provides an appropriate index within a latent modeling context, assessed the internal consistency of the seven-factor bifactor model (Raykov, 1998). Results of 0.60 and greater are considered acceptable (Diamantopoulos and Siguaw, 2000). The RPBS-Total demonstrated excellent internal consistency (ρ*c* = 0.96). Considering the subscales, the Traditional Religious Belief (ρ*c* = 0.84), Psi Belief (ρ*c* = 0.71), Witchcraft (ρ*c* = 0.78), Superstition (ρ*c* = 0.77), Spirituality (ρ*c* = 0.65), and Precognition (ρ*c* = 0.72) possessed satisfactory to good internal consistency. Composite reliability for Extraordinary Lifeforms was, however, lower than the threshold of 0.60 (ρ*c* = 0.46).

## Discussion

A comparison of 10 competing models of the RPBS found superior fit for a seven-factor bifactor solution. This solution comprised a general factor of paranormal belief alongside seven subfactors originally proposed by Tobacyk (1988). The factor loadings for all items but item 20 on the general factor were in the moderate to high range. In comparison, factor loadings were generally weaker for the seven subscales, but the majority of items loaded to an acceptable degree. This indicated that belief in the paranormal, as measured by the RPBS, is best characterized as a single overarching construct, comprising several related, but conceptually independent subfactors. This position is reassuring for previous work that has used the RPBS as a general (e.g., Dagnall et al., 2007) and/or multidimensional (e.g., Darwin et al., 2011) measure because it supports the notion that scores at both measurement levels are valid. Furthermore, although RPBS subscales contain only few items (three or four per factor) they possessed psychometric integrity and appeared theoretically consistent with their factor designation. The one exception being the Extraordinary Lifeforms factor, which demonstrated poor internal reliability (further discussion of this issue appears later).

At a factorial level, this study produced several important findings. Firstly, seven-factor solutions (Tobacyk and Milford, 1983; Tobacyk and Thomas, 1997) more appropriately represented RPBS content than five-factor iterations (Lawrence, 1995a; Lawrence and De Cicco, 1997; Lawrence et al., 1997). This outcome provides support for Tobacyk and Milford's (1983) original categorization of the RPBS and perhaps reflects greater similarities in phrasing among the items of the initial seven subfactors; a line of reasoning highlighted by the greater amount of error covariance required for the five-factor relative to the seven-factor solutions to achieve acceptable model fit. Secondly, oblique (Lawrence et al., 1997) solutions provided a better data-fit than orthogonal models (Tobacyk and Milford, 1983), suggesting that the specification of correlations between factors leads to meaningful improvements in model fit regardless of how many subfactors are incorporated.

Thirdly, bifactor models demonstrated superior fit for both five-factor and seven-factor solutions, supporting the existence of a general dimension of paranormal belief. This finding ran contrary to Tobacyk and Milford (1983), who concluded that rather than being a single dimension of belief in the paranormal there were several relatively independent paranormal dimensions. Studies that have used both the RPBS and the ASGS provide support for the notion that the RPBS adequately samples the paranormal belief domain; the two measures share approximately 60% shared variance (Drinkwater et al., 2012; Dagnall et al., 2014). This indicates that the RPBS as well as possessing construct breadth indexes the core aspects of the paranormal belief assessed by the ASGS (extrasensory perception, life after death and psychokinesis). Collectively, these results support a hierarchical conceptualization of paranormal belief, whereby a general paranormal belief factor relates to several specific belief dimensions. These data support this conceptualization, which represents a novel adjunct to the existing literature. Furthermore, women reported significantly higher levels of paranormal belief than men, which is a consistent finding in relation to previous research (e.g., Wolfradt, 1997). Support for invariance of the RPBS across gender indicates that mean differences in paranormal belief are unlikely to be artifacts of measurement bias, and rather suggest true mean differences.

Regarding the two-factor model, analysis revealed that the original seven subscales have contaminated RPBS purification. To illustrate this point, New Age Philosophy (NAP) derives almost exclusively from three original RPBS factors, Psi, Spirituality and Precognition, whilst Traditional Paranormal belief (TPB) consists of Traditional Religious Belief, Witchcraft and a single Psi item. In this context, it is clear that correlations present within the original seven-factor measure manifest within the two-factor model. This conclusion is consistent with Dagnall et al. (2017b), who found that it was necessary to covary errors among items belonging to the initial seven subscales to achieve satisfactory fit for both NAP and TPB. Other recent work utilizing the two-factor model has produced similar findings (e.g., Dagnall et al., 2017a).

At a conceptual level, the analysis supports previously expressed concerns about the Extraordinary Lifeforms (ELF) subscale and the use of a reversed item (question 23) to assess Psi. Considering these issues in turn, the authors are aware that critics contend that the inclusion of ELF is questionable because it is it is unclear whether belief in creatures, such as the Loch Ness monster, abominable snowman and extraterrestrials represent a paranormal dimension (Lawrence, 1995a; Dagnall et al., 2010). Rather than being beyond nature the existence of creatures, such as the Loch Ness monster and abominable snowman are improbable and elusive rather than supernatural. That is of course unless believers link them to paranormal powers or forces. To illustrate this point, researchers regularly discover new animal species (e.g., Ninja Lanternshark, Vásquez et al., 2015; Dusky Snout Catshark, Ebert and Clerkin, 2015).

Whilst, “paranormality” is an important concern the issue with the ELF scale was the relatively high endorsement rate of item 20, “There is life on other planets” (*M* = 4.88, *SD* = 1.61); this value indicates that respondents demonstrate moderate levels of agreement with this item. In comparison, respondents generally disagree with item 6, “The abominable snowman of Tibet exists” (*M* = 2.45, *SD* = 1.49) and item 13, “The Loch Ness monster of Scotland exists” (*M* = 2.39, *SD* = 1.52). Clearly, regardless of whether ELF items are paranormal, subscale content requires revision because it demonstrates poor internal reliability. This finding is consistent with the criticism that the extraterrestrial item is “useless” because most people regardless of general level of paranormal belief would agree with the statement that there is some form of life on other planets (Lawrence, 1995a). The authors do not support abandonment of the ELF subscale (see Lawrence et al., 1997), but instead recommend revision to ELF-item phrasing.

Concerning the Psi subscale, item 23 (“Mind reading is *not* possible”) loaded poorly on the factor. Scrutiny of the Psi means revealed that endorsement of the statement was high in comparison to other items (*M* = 3.87, *SD* = 1.90), which fell within a narrow range (*M* = 2.50, *SD* = 1.67 to *M* = 2.89, *SD* = 1.79). This finding was consistent with previous work, which reports that reversed items display lower reliability and weaker item-to-total correlations than positive-worded counterparts (Cronbach, 1942; Peabody, 1966; Benson and Hocevar, 1985). In addition to this, reversed items often prove difficult to accommodate within factorial models and frequently load on a separate factor (Benson and Hocevar, 1985; Pilotte and Gable, 1990; Herche and Engelland, 1996).

The current commonly used measures of paranormal belief (e.g., RPBS and ASGS) lack negatively keyed (reversed) items. Hence, within the RPBS, with the exception of item 23, endorsement of statements typically indicates belief in the paranormal. A frequently cited criticism of measures composed of predominantly positively framed items is that they incline respondents to answer in ways that do not reflect their actual view. Response bias is a major concern for scale developers because it can seriously compromise the validity of self-report scales (Van Sonderen et al., 2013). For example, clusters of unidirectional items will increase the tendency to agree or disagree to statements regardless of their content. Paradoxically, in the case of RPBS item 23 respondents often fail to notice the reversed wording of the statement as evidenced by the items poor psychometric performance.

Noting problems associated with response bias, scale developers recommend that scales comprise a balance of positively worded and reversed items (Baumgartner and Steenkamp, 2001). In the case of the assessment of belief in the paranormal generally and the RPBS specifically, the present study suggests that the use of reversed items may cause additional issues. Particularly, respondents often struggle to comprehend statements. Additionally, not believing in a specific instance/situation (mind reading) does not invalidate belief in ESP or telepathy. The question tells the researcher little about general belief in ESP; it is possible that respondents could believe that people have visions of the future, that people can communicate telepathically, see things remotely, but that they do not believe that information is transmitted via mental processes.

The RPBS despite being hierarchical and possessing construct breadth fails to reference important paranormal phenomena, such as ghost and poltergeists (Dagnall et al., 2010). These are important because both phenomena link closely to the survival hypothesis (e.g., life after death and spirits), which is a key paranormal concept. Additionally, belief in ghosts is high within contemporary society reflecting the significance of the topic (Gallup and Newport, 1990; Newport and Strausberg, 2001). Consequently, future scale developments and studies need to include items assessing belief in ghost and poltergeists (Dagnall et al., 2010).

Referencing the two-factor Rasch scaled model of Lange et al. (2000), it is important to note that this derived from a sample comprised of Australian adults. It would prove interesting to examine whether the gender and age biases observed within that population two-three decades ago apply to contemporary samples of other cultures, e.g., North American, British. This is an important point to consider for future research because beliefs and social attitudes evolve and change over time (Gergen, 1996). In addition, the current study did not perform further tests of reliability and validity including test-retest reliability and convergent validity. However, preceding research has supported temporal stability of the scale across a 4-week interval (Tobacyk, 2004). Future work, while assessing the latent structure of the RPBS, could also incorporate comparable measures of paranormal belief (e.g., the ASGS). This would provide a useful index of convergent validity.

Finally, it is worth noting that differences in sample size and composition may have contributed to the breadth of factorial solutions recommended previously. For example, several studies with relatively low respondent numbers drew exclusively on undergraduate student populations (Tobacyk and Milford, 1983; Lawrence et al., 1997). Clearly, future work would benefit from the use of larger more heterogeneous samples and the delineation of agreed expected sampling conventions and parameters. In conclusion, the current study indicates that a hierarchical latent structure, consisting of a general dimension of paranormal belief and seven conceptually independent subfactors best represents the RPBS. Strong factor loadings for a general factor and weaker, albeit acceptable, factor loadings for the subfactors supports the use of total RPBS scores and, to a lesser degree, subfactor scores within research. Findings also indicate that a seven-factor bifactor solution provides a robust conceptualization of the RPBS, evident by high reliability (alpha and composite) for all factors but Extraordinary Lifeforms.

## Author contributions

KD: theoretical focus and analysis; design, background and data collection. AD: theoretical focus and analysis; analysis and model testing. ND: theoretical focus and analysis; contributed to and supported all sessions. AP: commented on drafts—provided theoretical background and draft feedback.

### Conflict of interest statement

The authors declare that the research was conducted in the absence of any commercial or financial relationships that could be construed as a potential conflict of interest. The reviewer, GM, and handling Editor declared their shared affiliation.

## Footnotes

^1^AIC and ECVI for the two-factor oblique models were not compared with the other factor solutions due to possessing less RPBS items.
